# Advance Rectal Cancer in a Young Patient: Should Screening Start Early?

**DOI:** 10.7759/cureus.5195

**Published:** 2019-07-22

**Authors:** Swetha Parvataneni, Lionel Varela, Sireesha M Vemuri-Reddy

**Affiliations:** 1 Internal Medicine, Geisinger Health System, Lewistown, USA; 2 Family Medicine, Geisinger Health System, Lewistown, USA

**Keywords:** computed tomography (ct), magnetic resonance imaging (mri), cea- carcino-embryonic antigen, rectal cancer

## Abstract

Colorectal cancer is the third most common non-cutaneous malignancy in the United States, and the second most common cause of cancer-related deaths. Colorectal cancer is a broad term to include both colon and rectal cancer. Rectal cancer is commonly seen in age more the 50 years and often present with rectal bleeding. In this article, we will be discussing about a young female patient who presented with somatic pain as an initial symptom for metastatic rectal adenocarcinoma.

## Introduction

Rectal cancer is the most common malignancy of large intestine after proximal colon cancers [1]. Rectal cancer is commonly reported in patients over 50 years of age. In younger patients, rectal cancer is uncommon and mostly found in patients with positive family history. In the last decade, the incidence of rectal cancer has increased exponentially in patients less than 50 years, even without a family history of colorectal cancer [2,3]. The incidence of rectal cancer in younger population has increased annually by 3.5% in men, and 2.9% in women [4].

Rectal bleeding is the most common presentation for rectal cancer. Tenesmus, incomplete stool evacuation, rectal pain, and abdominal pain were also reported. Rarely bacteremia and peritonitis were noted because of perforation of the gastrointestinal lumen. If metastasis to lung or liver occurs, patients present with symptoms of the involved organ [5,6]. We will be discussing atypical presentation in a young patient for rectal cancer which was initially diagnosed as pyelonephritis prompting further evaluation.

## Case presentation

**Figure 1:**
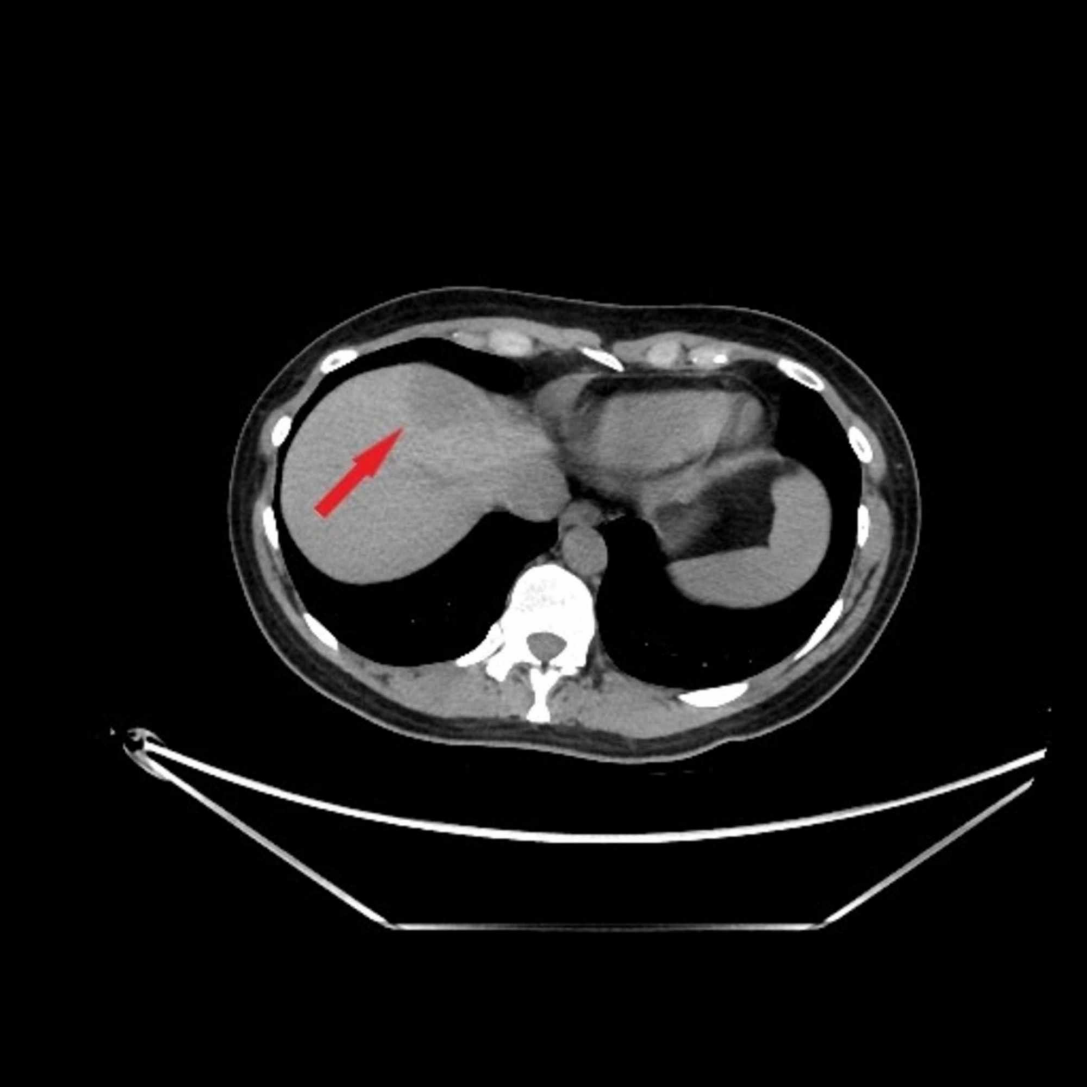
CT scan Hypo-attenuating lesion in the dome of the right hepatic lobe measuring approximately 2.6 x 3.2 x 2.6 cm.

A 30-year-old female with no significant past medical history or family history presented to the emergency room with left lower quadrant pain. She was treated symptomatically with follow-up. She was seen for follow-up by her primary care physician for flank pain, was treated with antibiotics, was sent for computed tomography (CT) of the abdomen and pelvis for clinical suspicion of pyelonephritis. The patient underwent CT scan which showed hypo-attenuating lesions in the right hepatic lobe (Figure 1).

**Figure 2:**
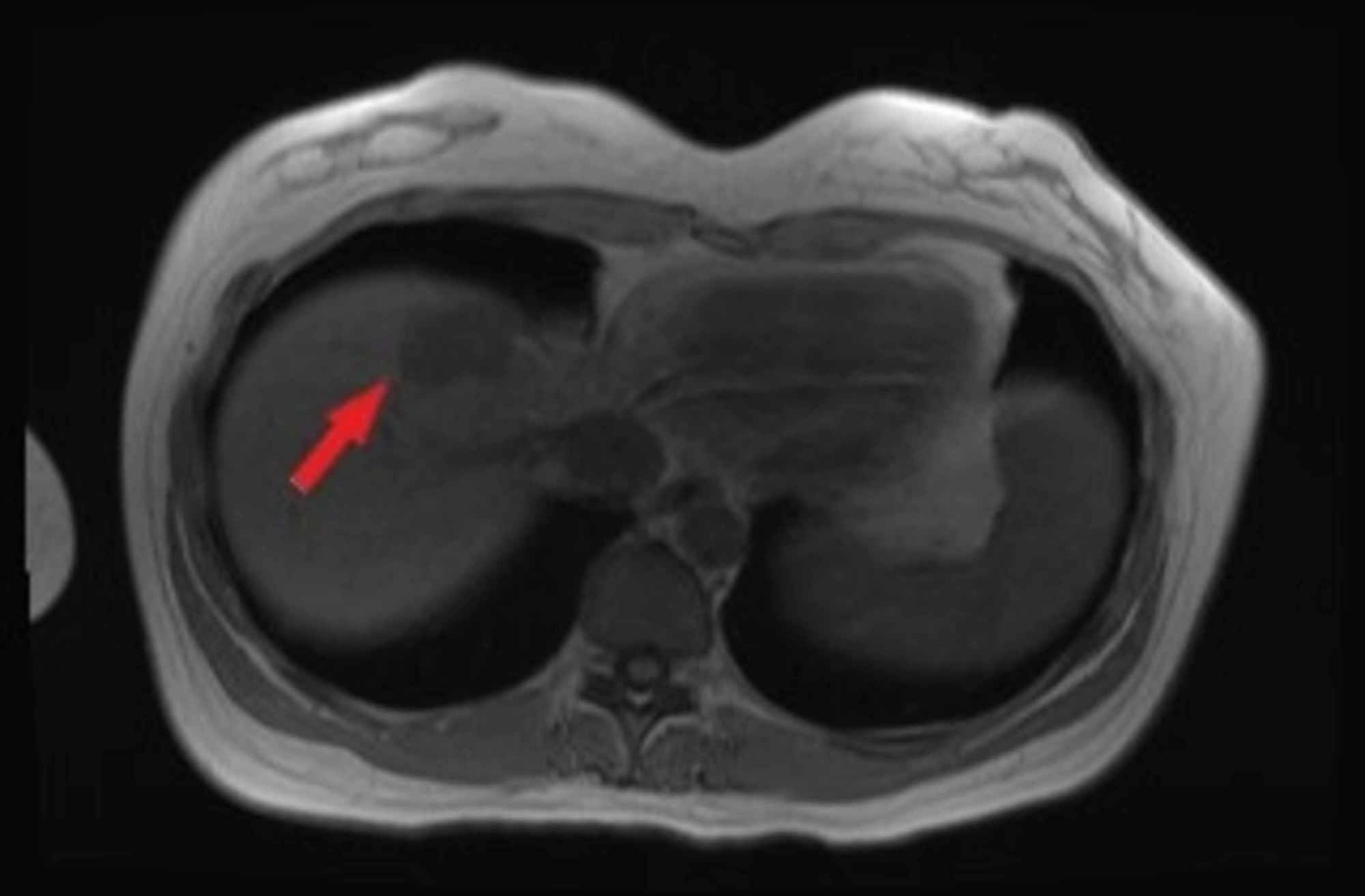
MRI Mass corresponds with hypo-attenuating lesion seen on CT scan.

The patient subsequently had magnetic resonance imaging (MRI) which showed lesions in the liver concerning for metastasis (Figure 2).

**Figure 3:**
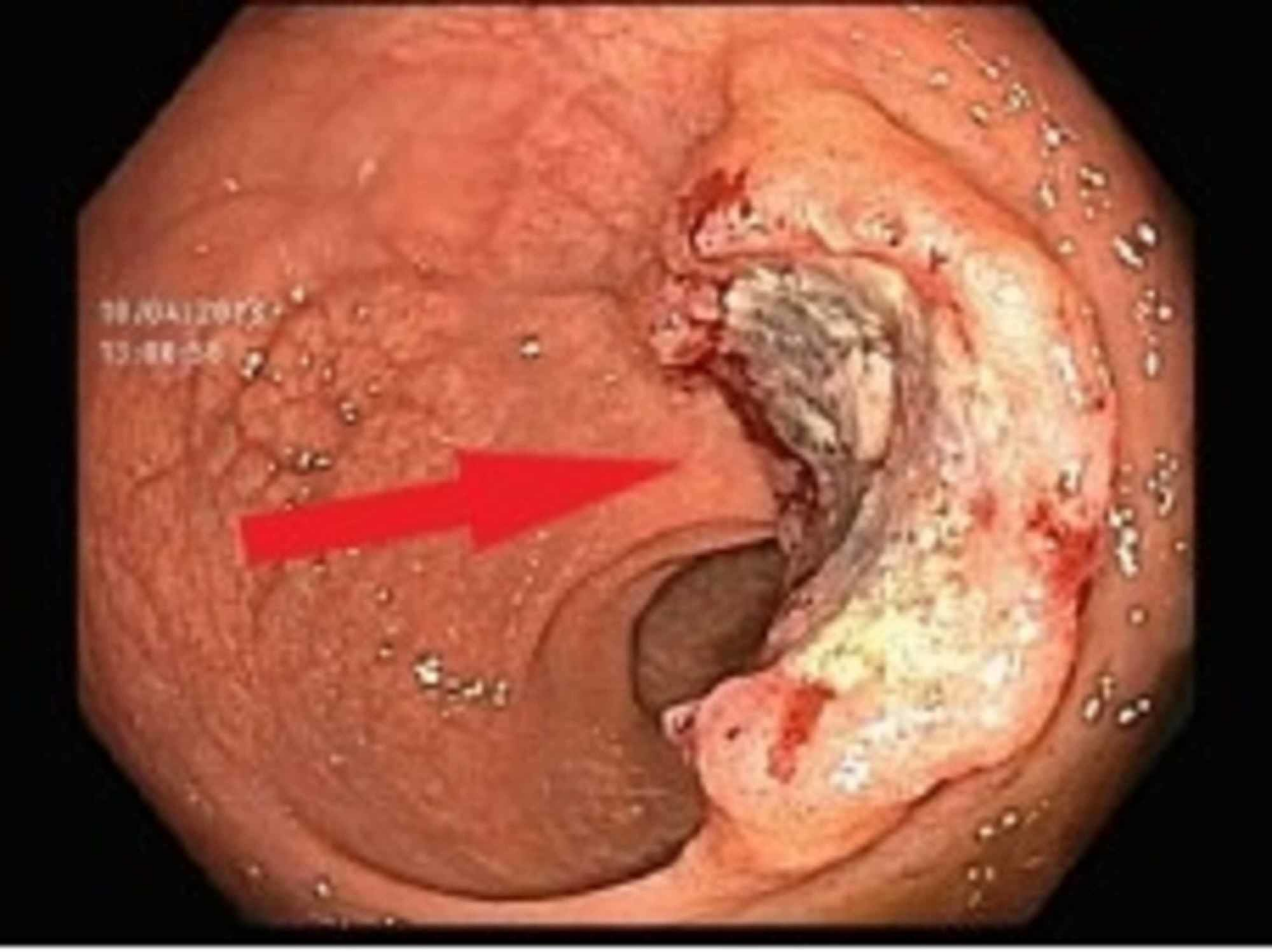
Colonoscopy Villous, fungating, infiltrative and deeply ulcerated non-obstruction large mass found in the rectum 10 cm from the anal verge.

The patient was referred to gastroenterologist and underwent colonoscopy. On colonoscopy, the patient was found to have rectal mass which was biopsied. She was diagnosed with rectal adenocarcinoma (Figure 3).

The patient underwent surgery and is currently receiving chemotherapy.

## Discussion

Rectum is the distal part of large intestine measuring about 12-15 cm. It is located between the sigmoid colon and anal canal. The wall of rectum has five layers: mucosa, submucosa, inner circular muscle, outer longitudinal muscle, and serosa. Tumor staging, assessment, and plan of surgery are based on cancer involvement of these layers. Both colon and rectal cancers have distinctive features because of their different embryonic origin. Rectal cancer originates in hind gut, and colon cancer in mid gut. Risk factors associated with both cancers also differ due to different areas of origin [7].

Multiple reviews and studies have described the risk factors for colorectal cancers. Family history of colon cancer, and personal history of adenoma are associated with increased risk of colon cancer compared to rectal cancer. Whereas, increased physical activity is associated with decreased risk of colon cancer. Elevated BMI is associated with increased risk of rectal cancer in women. High calcium, increased consumption of dairy products, and increased magnesium intake are associated with decreased risk of both colon and rectal cancers. Cigarette smoking and history of radiation therapy to prostate cancer increase the risk of rectal cancer compared to colon cancer [8-13].

Initial diagnosis of rectal cancer is clinical. Clinical suspicion of rectal cancer warrants further evaluation with imaging and colonoscopy. Sigmoidoscopy and colonoscopy are the preferred tests to diagnose rectal cancers. Although sigmoidoscopy can be used to diagnose rectal cancer, colonoscopy is indicated to examine rest of the large intestine, and detection of colorectal malignancies which co-exist with rectal cancer in 4-15% of cases [14]. Other non-invasive imaging modalities include double contrast barium enema, computed tomographic colonography (CTC), magnetic resonance imaging, and minimally invasive transrectal endoscopic ultrasound. CT scan has good sensitivity, and can be used as an alternative in the detection of colorectal cancers in patients refusing colonoscopy. MRI has 87% and 77% sensitivity respectively in detecting colorectal malignancy and staging the tumor [15,16]. Tumor markers such as carcinoembryonic antigen (CEA) and CA 19-9 aid in early diagnosis. After establishing the diagnosis, stage of the tumor determines need for surgery alone vs combined surgery and chemotherapy [17].

With the development of screening guidelines for colorectal cancer, early detection has led to decreased incidence of advanced colorectal cancers. Currently used screening modalities are colonoscopy, flexible sigmoidoscopy, CTC, fecal occult blood test, and fecal DNA. Routine screening is recommended at the age of 50 in average risk population or earlier in high risk population with positive family history. In the last decade, decreased incidence has been noted in patients > 50 years due to screening and early detection whereas, incidence has been increasing in patients < 50 years as currently screening guidelines do not recommend screening at this age without high risk factors. Younger patients present at later stages with metastatic disease, and have higher recurrence rates due to late detection. In the future, non-invasive modalities with adjusted screening guidelines need to be studied given increase in incidence and severity on presentation of rectal cancer in younger patients [3,7].

## Conclusions

Rectal cancer can present in advance stage as initial presentation in younger patients due to lack of screening and late detection. In future, studies are necessary to determine risk versus benefit for screening recommendations in patients less than 50 years after risk stratification. Less invasive screening modalities need to be developed in future which will help in early detection and diagnosis in the younger age group.
